# *C. elegans* Mutant Identification with a One-Step Whole-Genome-Sequencing and SNP Mapping Strategy

**DOI:** 10.1371/journal.pone.0015435

**Published:** 2010-11-08

**Authors:** Maria Doitsidou, Richard J. Poole, Sumeet Sarin, Henry Bigelow, Oliver Hobert

**Affiliations:** Howard Hughes Medical Institute, Department of Biochemistry and Molecular Biophysics, Columbia University Medical Center, New York, New York, United States of America; Brown University, United States of America

## Abstract

Whole-genome sequencing (WGS) is becoming a fast and cost-effective method to pinpoint molecular lesions in mutagenized genetic model systems, such as *Caenorhabditis elegans*. As mutagenized strains contain a significant mutational load, it is often still necessary to map mutations to a chromosomal interval to elucidate which of the WGS-identified sequence variants is the phenotype-causing one. We describe here our experience in setting up and testing a simple strategy that incorporates a rapid SNP-based mapping step into the WGS procedure. In this strategy, a mutant retrieved from a genetic screen is crossed with a polymorphic *C. elegans* strain, individual F2 progeny from this cross is selected for the mutant phenotype, the progeny of these F2 animals are pooled and then whole-genome-sequenced. The density of polymorphic SNP markers is decreased in the region of the phenotype-causing sequence variant and therefore enables its identification in the WGS data. As a proof of principle, we use this strategy to identify the molecular lesion in a mutant strain that produces an excess of dopaminergic neurons. We find that the molecular lesion resides in the Pax-6/Eyeless ortholog *vab-3*. The strategy described here will further reduce the time between mutant isolation and identification of the molecular lesion.

## Introduction

Whole-genome sequencing (WGS) is a new and powerful means to identify molecular lesions that result in specific mutant phenotypes [1], [2]. The WGS approach has been used in several studies in multiple model organisms, and our laboratory has successfully employed this strategy in the nematode *C. elegans* [3], [4], [5]. Sequenced genomes of mutagenized strains contain a substantial number of variants (on average more than 300 variants per chromosome, about 30 of which are in protein coding sequences) and a significant number of variants remain after outcrossing and/or may even be introduced by outcrossing [5]. Identification of the phenotype-causing mutation among the entire complement of sequence variants can be significantly facilitated by whole genome sequencing of two distinct alleles of the same locus. In such cases, little if any mapping is required since the phenotype-causing mutant locus will be one of the few, if not only loci found to be mutated in both WGS datasets. However, to avoid multiple WGS runs and/or if multiple alleles are not available, it may be desirable to first map the phenotype-causing variant to a chromosomal interval. The most commonly used mapping strategy in *C. elegans* employs single nucleotide polymorphism (SNP)-based mapping [6], [7], [8]. In this strategy, a mutant strain (usually derived from N2 Bristol) is crossed with a polymorphic Hawaiian *C. elegans* isolate and the mutant F2 progeny of such a cross are analyzed for their distribution of SNP markers. Genomic regions close to the mutation of interest show a decreased incidence of Hawaiian SNPs while unlinked regions contain an even representation of Hawaiian vs. Bristol SNPs. However, this conventional SNP mapping strategy usually starts with a relatively small, arbitrarily chosen number of SNPs. Through re-iterative SNP mapping to finer and finer regions, the gene can be fine-mapped, but this can be a relatively tedious and time-consuming process. We describe here the employment of a strategy - modeled on a similar strategy (“SHOREmap”) previously employed in plants [9] - that combines WGS with a very fine-grained SNP mapping strategy in a single step. This combination significantly reduces the time between mutant isolation and identification of its molecular identity.

## Results

### Overall strategy

**Figure 1:**
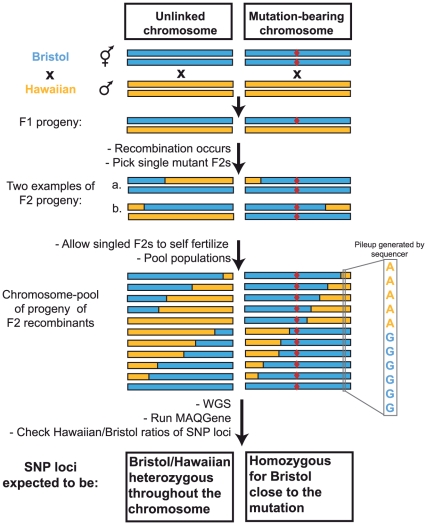
Principle of the WGS-SNP strategy. The red diamond indicates the mutation of interest. The sequence ‘pileup’, generated by processing the WGS sequencing data, is a representation of the number of reads that are mapped to a specific position in the genome. The relative number of N2 vs. Hawaiian nucleotides is a reflection of the relative distribution of recombinants in the pool.

The overall strategy is schematically depicted in **Fig. 1**. A mutant strain, for which no mapping information is available, is crossed with the polymorphic Hawaiian strain CB4856 [10] and a number of F2 progeny that carry the mutant phenotype are singled onto fresh plates. Each of the isolated F2 animals represents a recombinant in which the Hawaiian chromosomes have recombined with wild-type, Bristol-derived chromosomes. The progeny of these singled F2 hermaphrodite animals ( = F3 and F4 generation) are then pooled, DNA is prepared and the entire pool is subjected to WGS.

Due to meiotic recombination, in regions unlinked to the mutation the parental chromosomes will recombine in a largely non-biased manner. So as long as enough recombinants are pooled, unlinked SNP loci will appear in a roughly 50/50 ratio of Hawaiian vs. Bristol nucleotides in the sequence output pileup generated by the genome sequencer (**Fig. 1**). In contrast, the closer a SNP locus is to the mutation, the more rare it is to find a recombination event between that SNP and the mutation (**Fig. 1**). As a result, Hawaiian variants in the sequence pileup will be underrepresented in regions closer to the selected mutation. Finally, we expect to have only Bristol sequences in the sequence pileup very near the mutation. Identifying an extended region of pure Bristol sequence in such an approach is meaningful, since Hawaiian SNPs are uniformly distributed across all chromosomes with an average density of about 1/1,000 [11].

The WGS dataset is analyzed using a software tool that we recently developed, called MAQGene [12]. We modified MAQGene to now perform two different functions: (i) it performs the conventional function of identifying homozygous variants of non-SNP loci between the mutant strain and the reference Bristol genome; (ii) it now also considers the ratios of Hawaiian vs. Bristol representation in the pileup at all known SNP loci in the genome (∼100,000 variants)[11]. Therefore, the same dataset originating from a single WGS run for a given mutant will not only reveal the SNP distribution but will also greatly improve the ability to identify the phenotype-causing mutation, which will be one of the few -if not the only- variants in an interval defined by the SNP mapping.

A key advantage of this strategy is that rather than examining an arbitrarily chosen, limited number of SNPs to assess the map position of a specific mutation (usually at most a few dozen [6]), this strategy interrogates in a single step, at least in theory, all ∼100,000 SNPs that distinguish the Hawaiian *C. elegans* isolate from the Bristol isolate. Therefore, the WGS-SNP combination not only eliminates all technical aspects of SNP mapping (PCR, sequencing or restriction mapping of PCR amplicons), but also provides, in one step, a much finer grained resolution. Moreover, it reduces the costs of mutant identification, since it employs a single whole-genome sequencing run for both mutant mapping as well as mutant identification.

### Proof of principle

**Figure 2:**
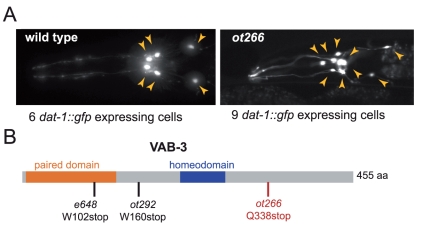
*ot266* mutant animals show ectopic expression of a dopaminergic cell fate marker. **A:** The head region of an adult worm is shown. 4 CEP and 2 ADE neurons express *gfp* in wild-type animals. The marker is *vtIs1* (*dat-1::gfp*). See Table 1 for quantification. **B:** VAB-3 protein structure and location of alleles.

We used this strategy to identify the molecular lesion in a previously uncharacterized mutant strain that carries the *ot266* allele. *ot266* is a recessive mutation that we isolated in a screen for mutants with defective dopaminergic neuron specification (“Dopy:” phenotype), as assessed by expression of the dopaminergic marker *dat-1::gfp* (expressed from the transgene *vtIs1*)[13]. *ot266* mutants display an aberrantly increased number of dopaminergic neurons (**Fig. 2A**; **Table 1**). We crossed *ot266*; *vtIs1* animals with the Hawaiian CB4856 strain and singled F2 progeny with the Dopy phenotype. To assess how many recombinants are required to yield reliable results, we pooled in one experiment the progeny of 20 F2 animals and in another experiment the progeny of 50 F2 animals. The pooled DNA from each experiment was then subjected to WGS on an Illumina GA2 sequencing platform, using single end, 100 nucleotide reads. One or two lanes of a flow cell were used to assess the effect of various sequencing depths on mapping resolution (**Table 2**).

**Table 1:** Quantification of the Dopy phenotype.

Genotype	Additional *gfp-*positive cells 1	1 additional *gfp-*positive cell	2 additional *gfp-*positive cells	3 additional *gfp-*positive cells	n
Wild type	0%	0%	0%	0%	>100
*vab-3(ot266)*	88%	49%	28%	9%	43
*vab-3(ot292)*	76%	53%	18%	2%	51
*vab-3(e648)*	88%	40%	21%	17%	48
*vab-3(RNAi)* 2	20%	17.5%	2.5%	0%	40

1 Percentage of animals with ectopic *dat-1::gfp* (*vtIs1)* expression.

2 RNAi was performed in a genetically sensitized strain background, *eri-1*; *lin-15b* [17] (see Material and Methods).

**Table 2:** WGS mapping results.

# of pooled recom-binants	# of lanes used for data analysis	Coverage across all non-gap regions	Defined mapping interval (nucleotide position according to Wormbase release WS201)	Defined mapping region in Mb	Defined mapping region in map units	# of protein changing variants in the region
50	2	41x	8841415–10975250 (0.43 to 2.77 cM)	2.13	2.34	3
50	1 (lane #1)	20x	8908053–10975250 (0.44 to 2.77 cM)	2.06	2.33	3
50	1 (lane #2)	20x	8841415–11698279 (0.43 to 6.16 cM)	2.85	5.70	7
20	1	18x	6056881–10981227 (−3.78 to 2.78 cM)	4.90	6.56	9

**Figure 3:**
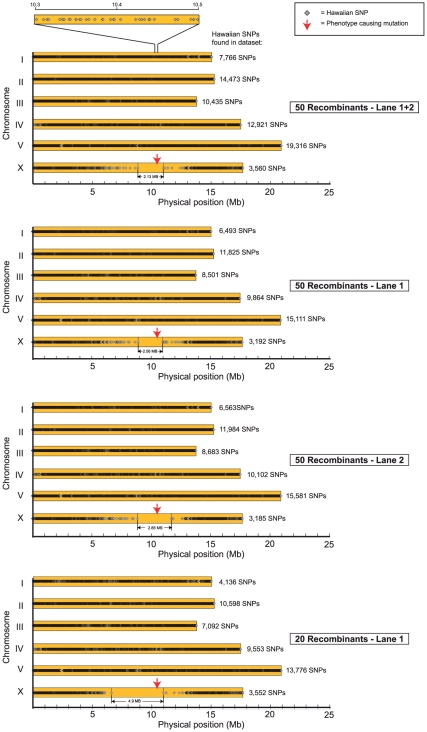
Application of the WGS-SNP strategy for mapping and cloning *ot266*. Red arrow indicates the phenotype-causing mutation in *vab-3*(*ot266*). Numbers next to the chromosomes depict how many SNP loci show occurrence of Hawaiian SNPs in the 0.2–0.6 range (see Material and Methods for explanation of this range). Note that the mapping interval obtained from the 50 recombinants, lane 1 dataset is smaller than the one defined from 50 recombinants, two lanes dataset (see also **Table 2**). We believe that this is due to random variability in sample representation in the flow cell. Also note that this dataset does not allow to easily infer the position of the *vtIs1* transgene since we did not select for homozygous *vtIs1* animals in the F2 generation.

**Figure 4:**
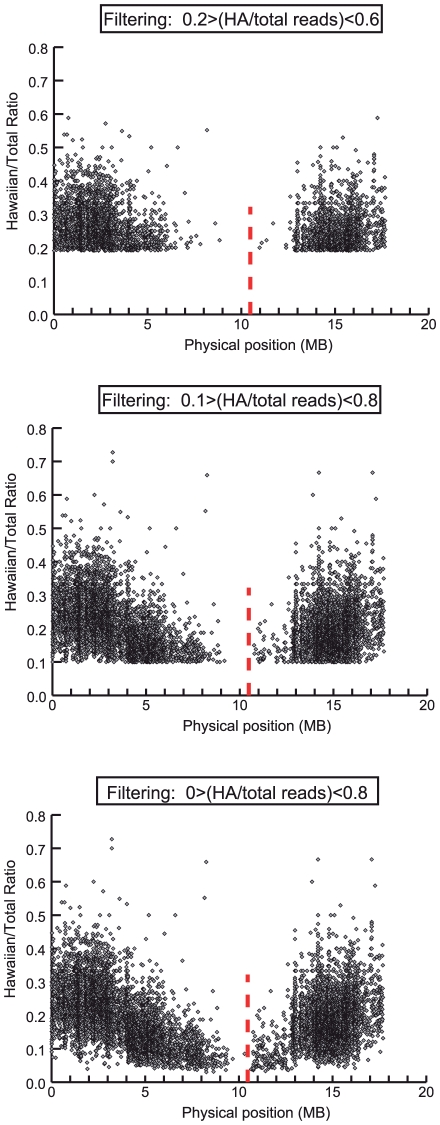
Alternative graphic representation of the WGS-SNP data. The WGS SNP data is shown for Chromosome X, 50 recombinants, 2 sequencing lanes. The positions of SNP loci are depicted as a XY scatter plot, where the ratio ‘Hawaiian/total number of reads for each SNP is represented. The red line indicates the position of the phenotype-causing variant. 3 different filtering criteria were used, as indicated in the figure. Note that the non-linear appearance of the right arm of the scatter plot reflects the increased recombination rate on that part of the chromosome (as can be visualized by Marey map for CHR X –data not shown). Using 0.1–0.8 filtering criteria, narrowed down the mapping interval even further (1.57 Mb).

As mentioned above, we modified MAQGene to provide an additional output file with the sequence pileup at all Hawaiian/Bristol polymorphic positions and the relative frequency of Hawaiian and Bristol sequences at these positions. We extracted from the list of SNPs those loci that had sufficient coverage and whose pileup showed a defined proportion of Hawaiian sequences (for the exact filtering criteria and data processing see Materials and Methods). Graphs showing the distribution of those SNPs on the 6 chromosomes were then generated. We expected the mutation-bearing region would be revealed by the lack of such Hawaiian SNPs. The results are depicted in **Fig. 3** and **Table 2**. An alternative graphic representation of the data is shown in **Fig. 4**.

Through the dip in the distribution of Hawaiian SNPs, the “50 F2 pooled” dataset (2 lanes sequenced) defined a mapping interval of 2.1 megabases (Mb) or 2.34 map units on the X chromosome (**Fig. 3** **;** **Table 2**). To test the effect of coverage on the resolution of the method, we analyzed the data from each of the two lanes of the flow cell, one at a time. These datasets define a mapping interval of 2.06 Mb for one of the two lanes and 2.85 Mb for the other (**Fig. 3**; **Table 2**; the reason for these interval sizes being different may be a consequence of differential sample representation in the flow cell). The sequence data from the progeny of 20 F2 recombinants (1 lane sequenced) defined a mapping interval of 4.9 Mb (**Fig. 3** **,** **Table 2**).

We then used the MAQGene variant output file to find the homozygous Bristol variants that fall in the defined mapping interval (see Materials and Methods for variant filtering). The smallest interval defined by SNP mapping (2.06 Mb) contained only 3 protein-coding sequence variants (**Table 2** and **Table S1**). One of the three variants is a premature stop codon (Q338Stop) in the *vab-3* locus (**Fig. 2B**), which codes for the sole *Pax6/Eyeless* homolog of *C. elegans* [14]. Our genetic screen uncovered one more allele with a similar Dopy phenotype (*ot292*)(**Table 1**) and the same morphological abnormalities as *ot266* animals. Manual Sanger-sequencing of that additional allele also identified a premature stop codon in the *vab-3* locus (W160Stop)(**Fig. 2B**). In addition, the *ot266* phenotype is recapitulated by the *vab-3* reference allele *e648* and by knockdown of *vab-3* with RNAi (**Table 1**). We conclude that *vab-3* is involved in controlling the generation of dopaminergic neurons. We note that a third, previously identified *vab-3* allele also shows a Dopy phenotype, yet unlike the alleles described here, this allele has an additional phenotype, i.e. partial loss of DA neurons [13].

## Discussion

We have successfully applied a SNP- and WGS-based cloning strategy for mutant identification in *C.elegans*. This strategy has already been successfully employed in *Arabidopsis thaliana* [9]. For the *Arabidopsis* study, 500 recombinants were used and sequencing was done with single run reads to a coverage of 22x. Our testing of this strategy has identified a tight and cost-effective parameter set for the use of this approach in *C.elegans*. We find that analyzing 10-fold fewer (50) homozygous recombinant pools provides fine-grained mapping information. In the case presented here, it provided a ∼2 Mb mapping interval and limited the candidate phenotype causing mutations to only 3 protein changing variants. Even the analysis of as few as 20 homozygous recombinants allowed for mapping of the mutation to a <5 Mb interval with 8 protein-changing variants (compared to an average of 130 protein-changing variants found all across mutagenized genomes). As recombination frequency is not uniform throughout the genome [15], the number of recombinants needed to obtain mapping to a few megabases will differ on a case by case basis, but we consider 20–50 recombinants a reasonable compromise between amount of effort needed and mapping resolution.

We also find that the sequencing depth that we currently employ for standard *C.elegans* WGS (1 lane of a flow cell, non-paired 100 nucleotide reads) provides sufficient data to query the genome for Bristol/Hawaiian SNPs. The approach presented here can also be used to map a dominant mutation. To this end, one would need to test which of the individually isolated F2 animals are homozygous for the mutation by examining their F3 progeny. Only homozygous F2 animals shall be processed further. The method can also be used to map a phenotype-causing deletion [5].

Recently, Zuryn et al. provided a proof-of-principle study for another approach to map and clone genes within a single WGS run [16]. Rather than crossing a mutant *C. elegans* strain to a polymorphic mapping strain, the authors backcrossed their mutant strain with the starting strain used for mutagenesis. Backcrossing several times eliminated all unlinked mutagen-induced sequence variants, while linked, mutagen-induced variants remained, thereby providing mapping information [16]. The Zuryn et al. strategy requires multiple, non-allelic *C. elegans* strains to eliminate background variants through comparing WGS datasets [16]. The WGS-SNP-based strategy described here does not involve comparing different WGS datasets (and therefore works with individual mutants), does not require the availability of the starting *C. elegans* strain for outcrossing, and it requires only a single cross. However, it does require the availability of a reasonably well-characterized polymorphic strain, such as the Hawaiian CB4856 strain.

As pointed out before [9], the combined SNP/WGS strategy that we describe here for *C. elegans*, can of course be applied for mutant identification in many other model system species in which positional cloning has traditionally been tedious.

## Materials and Methods

### Strains

The following strains were use: Hawaiian strain CB4856 [10], BY200 *vtIs1[dat-1::gfp*; *rol-6(d)]*, OH4254 *ot266*; *vtIs1[dat-1::gfp*; *rol-6(d)]*, OH4330 *ot292;vtIs1[dat-1::gfp;rol-6(d)]*, CB648 *vab-3(e648)*. RNAi was performed on a sensitized background strain, OH9315 (*eri-1*; *lin-15b*; *vtIs1*).

### Genetic screen and RNAi

OH4254 animals were mutagenized with EMS and were kept at 25°C at all times. 5 parental (P0) mutagenized animals were placed in each of 10 founder P0 plates. Three days later, 400 F1 progeny of the mutagenized P0 animals were singled. Their ensuing progeny (F2 and F3 generation) were screened under a stereomicroscope equipped with a fluorescent light source and individual mutant animals were picked to establish mutant lines. For recapitulating the *vab-3* phenotype with RNAi we plated L3 stage, *eri-1*; *lin-15b*; *vtIs1* worms on plates coated with bacteria expressing a *vab-3* dsRNA clone and scored the F1 progeny for *gfp* expression from the *vtIs1* transgene.

### Mapping cross and DNA preparation

We crossed *ot266* animals with Hawaiian CB4856 males and singled F1 cross progeny. In the F2 progeny, we picked 20 (or 50) progeny with the Dopy phenotype and singled them onto individual plates. We allowed the F2 recombinants to self fertilize until they filled 6 cm plates. We pooled the progeny of 20 (or 50) F2 animals, using approximately similar numbers of animals from each plate. The pooled worms were lysed and genomic DNA was prepared according to the standard protocol used for sample preparation for WGS (http://biochemistry.hs.columbia.edu/labs/hobert/protocols.html). DNA libraries were prepared according to Illumina’s protocol. The pooled DNA from each experiment was then subjected to WGS on an Illumina GA2 sequencing platform, using single 100 nucleotide reads.

### Data analysis with MAQGene

To run MAQGene we used the general parameters previously described [12] setting “max sum of error qualities for mapping assembly and pileup” to 200. To call a variant we used the following general criteria: loci multiplicity < = 1, total sequencing depth > = 3, consensus quality score > = 3, neighboring score > = 3, and fraction of non-wt reads > = 0.8.

For identifying the mapping interval, MAQGene was modified to generate an additional output file that provides a pileup for all annotated SNP loci, the number of Bristol reads, the number of Hawaiian reads and the sequencing depth. This file contains all annotated SNPs in the Bristol genome and needs to be filtered using, for example, Microsoft Excel to include only Hawaiian SNPs (named as haw). Using this file we calculated the ratio of Hawaiian (HA) reads in Excel (ratio of HA reads =  number of HA reads/total number of reads). To minimize the chance that random sequencing errors would falsely appear as HA sequence in SNP loci, we did not consider loci with very low occurrence of HA sequences. In particular, we filtered the dataset to include only SNPs with at least 3 HA reads and a HA ratio of at least 0.2. In addition, we did not include in our analysis SNPs with ratios of HA sequences above 0.6. The reason for this upper ‘cutoff’ is the existence of loci annotated as SNPs in the Bristol genome that we actually found to be (in multiple Bristol based sequenced strains) identical to the Hawaiian annotated sequence for this locus. Thus, these loci (a few hundred in total) are ‘falsely’ annotated SNPs, possibly representing Bristol sequencing errors or loci of heterozygosity and are not indicative of the actual existence of Hawaiian sequences in a region. We excluded these loci from our datasets by filtering out everything that appeared too high on Hawaiian ratios (above 0.6). We have compiled a list with SNP loci that in two or more of our Bristol datasets show HA sequence (**Table S2**).

We then checked the distribution of SNPs that fulfilled the above filtering criteria across the six chromosomes. We did that by using an Adobe Illustrator XY scatter plot to graphically represent for each chromosome the physical location of each SNP (**Fig. 3**). Chromosome X was immediately revealed as the mutation-bearing chromosome from the existence of an extensive region devoid of HA SNPs (**Fig. 3**). The mapping region was defined by the physical location of the two consecutive HA SNPs located the furthest apart from each other. As an alternative way of representing the data, we generated an XY scatter plot of the position of the SNPs in which the ratio of HA sequence representation over the total sequencing depth is also depicted (**Fig.4**). For this plot, the same general filtering criteria described above were used.

For identifying the molecular lesion of the mutant, we filtered in Microsoft Excel the variant MAQGene output file for protein changing variants. In particular we filtered the class of mutations to fulfill the following conditions: [does not contain ‘SNP’] AND ([contains ‘missense’] OR [contains ‘stop’] OR [contains ‘donor’] OR [contains ‘acceptor’]).

We did not filter out common variants that appear in our previously sequenced datasets as the resolution of this method does not require comparison with additional datasets.

### MAQGene modification

The aforementioned new SNP-specific output file contains one line per known SNP location with the following fields: chromosome, position, snp name, snp base, number of snp-containing reads, reference base, number of reference-containing reads, total number of reads.

In order to produce this extra results file, we added one step to MAQGene's database building part and one to the pipeline itself. The database building step is necessary because the previous version, which extracts all of its annotation information from ftp.wormbase.org/pub/wormbase/genomes/c_elegans/genome_feature_tables/GFF3/c_elegans.WS201.gff3.gz lacks actual SNP base for each listed SNP. The new step additionally extracts information from ftp.wormbase.org/pub/wormbase/genomes/c_elegans/annotations/WS201_elegans_SNP_changes.txt.gz, which provides this information.

During the pipeline itself, the pileup file (already produced routinely) is scanned a second time to extract all positions of known SNPs, regardless of low sequencing coverage or other filters that are applied in the main results file. Together with the new information about actual SNP base, the number of reads matching either the SNP base or reference base are counted and reported in a separate tabular text file.

As usual, users interested in this extra feature must update their installation of MAQGene and re-run the database building step (build_annotation_tables.sh).

## Supporting Information

Table S1 List of mutations in the mapping region. (DOCX)

Table S2 Bristol SNP loci with Hawaiian sequence. (XLS)
