# Supplementary material for: C. elegans Mutant Identification with a One-Step Whole-Genome-Sequencing and SNP Mapping Strategy
Source: PLoS One. 2010 Nov 8;5(11):e15435. doi: 10.1371/journal.pone.0015435 (PMC2975709; doi:10.1371/journal.pone.0015435)
Supplement: Table S1 — List of mutations in the mapping region. (DOCX) [file pone.0015435.s001.docx]

**Table S1: List of mutations in the mapping region**

50 recombinants, 2 lanes:

| CHR | start | reference_base | sample_base | classes | parent_features |
| --- | --- | --- | --- | --- | --- |
| X | 9407197 | C | A | premature_stop | C23F12.2 |
| X | 10482425 | C | T | missense | C33D3.1 |
| **X** | **10517579** | **C** | **T** | **premature_stop** | **F14F3.1a,F14F3.1b,F14F3.1c** |

50 recombinants, lane #1:

| CHR | start | reference_base | sample_base | classes | parent_features |
| --- | --- | --- | --- | --- | --- |
| X | 9407197 | C | A | premature_stop | C23F12.2 |
| X | 10482425 | C | T | missense | C33D3.1 |
| **X** | **10517579** | **C** | **T** | **premature_stop** | **F14F3.1a,F14F3.1b,F14F3.1c** |

50 recombinants, lane #2:

| CHR | start | reference_base | sample_base | classes | parent_features |
| --- | --- | --- | --- | --- | --- |
| X | 9407197 | C | A | premature_stop | C23F12.2 |
| X | 10482425 | C | T | missense | C33D3.1 |
| **X** | **10517579** | **C** | **T** | **premature_stop** | **F14F3.1a,F14F3.1b,F14F3.1c** |
| X | 11077741 | C | T | missense | W04G3.10 |
| X | 11375072 | G | A | premature_stop | F42E11.3 |
| X | 11660042 | C | T | missense | T04F8.1 |
| X | 11695504 | C | T | missense | C44C10.4 |

20 recombinants, one lane

| CHR | start | reference_base | sample_base | classes | parent_features |
| --- | --- | --- | --- | --- | --- |
| X | 6383443 | C | T | missense | SSSD1.1 |
| X | 6792031 | G | C | missense | H22K11.1 |
| X | 7588883 | T | C | missense | F08C6.1a.1,F08C6.1a.2,F08C6.1c |
| X | 8033820 | G | C | missense | C18A11.6 |
| X | 8292726 | C | T | missense | F13B9.1a,F13B9.1b,F13B9.1c |
| X | 9407197 | C | A | premature_stop | C23F12.2 |
| X | 10482425 | C | T | missense | C33D3.1 |
| **X** | **10517579** | **C** | **T** | **premature_stop** | **F14F3.1a,F14F3.1b,F14F3.1c** |
